# Beyond the reckoning: Addressing structural anti-Black racism in population and public health

**DOI:** 10.17269/s41997-025-01093-7

**Published:** 2026-01-05

**Authors:** Sume Ndumbe-Eyoh

**Affiliations:** Black Health Education Collaborative, Dalla Lana School of Public Health, University of Toronto, Toronto, ON Canada

**Keywords:** Racism, Black health, Anti-racism, Health equity, Racisme, Santé des personnes noires, Antiracisme, Équité en santé

## Abstract

The absence of historical context in public health has contributed to the persistent neglect of structural racism as a major determinant of health inequities. This commentary critically highlights the omissions of the Lalonde Report, a foundational document in population health, and explores the enduring impacts of slavery, colonialism, and systemic racism on Black health in Canada. Anti-Black racism continues to shape racial health inequities through economic, political, and social marginalization. However, public health research, policy, and practice have largely failed to address the structural dimensions of racism. Yet, Black communities in Canada have long resisted these injustices through grassroots movements, community advocacy, and systems transformation. A decolonial, anti-racist approach is necessary to disrupt these patterns, shift power and resources, and promote health equity. This requires interdisciplinary education, policy change, and investments in social systems that promote equity for Black communities. Centring community-led solutions, institutional accountability, and leveraging legislative tools are critical steps toward meaningful change. Public health must actively engage in dismantling white supremacy to achieve equitable health outcomes for Black communities.

## Résumé

L’absence de contexte historique en santé publique contribue au désintérêt persistant pour le racisme structurel en tant que déterminant majeur des iniquités en santé. Notre commentaire critique les omissions du rapport Lalonde, un document fondamental en santé des populations, et explore les effets durables de l’esclavage, du colonialisme et du racisme systémique sur la santé des personnes noires au Canada. Le racisme anti-Noirs continue de structurer les iniquités raciales en matière de santé par le biais d’une marginalisation économique, politique et sociale. Dans une large mesure toutefois, la recherche, les politiques et les pratiques en santé publique n’abordent pas les dimensions structurelles du racisme. Néanmoins, par les mouvements populaires, le plaidoyer communautaire et la transformation des systèmes, les communautés noires du Canada résistent depuis longtemps à ces injustices. Une approche décoloniale et antiraciste est nécessaire pour perturber ces schémas, déplacer le pouvoir et les ressources et promouvoir l’équité en santé. Elle exige une formation interdisciplinaire, un changement de cap et des investissements dans des systèmes sociaux qui favorisent l’équité pour les communautés noires. Un recentrage sur les solutions communautaires, une responsabilisation des institutions et l’utilisation d’instruments législatifs sont les étapes essentielles d’un véritable changement. La santé publique doit activement participer au démantèlement de la suprématie blanche pour obtenir des résultats équitables pour la santé des communautés noires.

## The absenting of history in population health

The absence of history in public health is a longstanding concern, particularly related to addressing racial health inequities. The Lalonde Report, one of the most influential contributions to population health, disregarded the pervasive role of systemic racism and interrelated systems of dispossession in shaping health inequities. This absence, mirrored in most public health, has negative impacts for Black communities in Canada. This commentary revisits the Lalonde Report’s omissions, integrating historical analysis, contemporary evidence, and strategies for transformative change. A decolonial anti-racist paradigm in health promotion and public health, that is attentive to the historical and contemporary manifestations of racism, is needed to actively disrupt the ways in which colonialism, enslavement, and systemic racism continue to harm health.

### Slavery in Canada: Looking back to move forwards

The history of slavery in Canada is often ignored, yet it is essential to understanding the systemic dehumanization of Black people and contemporary health inequities (Maynard, 2017). Chattel slavery, which was a legitimate enterprise in Canada, created the context for the racialized exploitation of African people globally. Through the creation of racial hierarchies, Black people were constructed as subhuman. This justified the subsequent displacement, dispossession, and exploitation and normalized the ensuing inequities. Slavery ended in 1834 in Canada and the rest of the British Empire. This legacy, not even 200 years old, has disappeared from Canadian public health narratives despite its enduring impacts on health and well-being for Black communities.

In refusing an ahistoric positioning, we remember that Canada, the settler colony that it is, included many slave holders in its first and second parliaments. Furthermore, notable health institutions like McGill University’s Faculty of Medicine were founded by people who were slave holders and public health was implicated in the destruction of Africville, a historic African Nova Scotian community. This historical experience of racism sets the stage for Black health inequities today.

## Racism as the driver of racial health inequities

Racism is a violent system of power that causes racial health inequities that are felt deeper by those at the nexus of multiple systems of harm (Public Health Agency of Canada, 2020). Ruptures to land-based relationships through the violence of (settler) colonialism and enslavement have created deep health inequities for Black, Indigenous, and racialized communities. Racism profoundly impacts health and well-being by creating and perpetuating economic, political, and social inequality in health-promoting resources throughout the lifecourse. For example, limited access to adequate income, decent jobs, housing, and education, along with exposure to toxic environments and chronic racial trauma, worsens health outcomes for Black communities. Converging crises of white supremacy and the climate crisis disproportionately impact Black communities globally.

### Structural racism, racial inequities, and Lalonde, or did racism exist in 1974?

The Lalonde Report was instrumental in shifting the discourse and practice of health from a focus on biomedical drivers of health to a broader consideration of social and economic contexts that influenced health. Despite Canada’s deep entanglements with structural racism, anti-Blackness, and colonization, the Lalonde Report (Lalonde, 1974) reflects the tradition of absenting the critical role of structural racism as a major driver of racial health inequities, a trend which continues to be evident in public health research, policy, and practice. Published only 5 years after Africville was destroyed following systemic neglect, this complete silence is troubling. Importantly, the demolition of Africville is part of a pattern of neglect of Black communities. In Canada, opportunities to develop meaningful state and public health responses to structural racism and anti-Black racism have often fallen short, largely because racism is not seen as a priority in policy making domestically or internationally (Banting & Thompson, 2021). To date, Canada has abstained from signing on to the Durban Plan of Action on Racial Discrimination, which remains the major international instrument on racial discrimination. Yet, racial health inequities persist, and in some instances are worse than they were before the COVID-19 pandemic. Still, public health research, policy, and practice continue to (1) downplay the impact of racism on health, and (2) focus on downstream approaches instead of upstream/structural interventions.

## Thriving through radical love and imagination

### Resistance

Against this backdrop, there have been Black presence and resistance in this part of Turtle Island for centuries. African presence in the Americas predates slavery and colonization, and Black people have lived in Nova Scotia since the early 1600s, which remains home to over 50 historic African Nova Scotian communities. Resistance movements have been a central part of the Black experience in Canada. For example, Marie Joseph Angelique, a Portuguese-born black woman, defied her enslavement and was hanged in 1734 after being accused of burning down present-day old Montreal. Organizations like the Black United Front provided health, social, and legal supports to communities, including protection from police brutality. The Black Cross Nurses organized to address health promotion and demand the inclusion of Black people in nursing training. Today, continued resistance to structural racism, amplified by movements like Black Lives Matter, is shifting the landscape for political and policy action.

Following Black feminist traditions, an ethic of radical love calls on us to design societies that uphold liberation, equity, and community-building as counterpoints to systems of oppression that allow for all to thrive (hooks, 2003). Dismantling systemic racism demands troubling (settler) colonialism, structural white supremacy, and anti-Blackness, and amplifying movements for Indigenous sovereignty and Black liberation. Taking a decolonial stance to all aspects of public health requires interrogating our complicity in maintaining racial inequities. It, “means working to undo in insidious mechanisms of colonialism that have penetrated our ways of knowing, being, and doing, and shaped the stories we tell about ourselves, each other, and what equity might look like between us” (Allan, 2022, pp. 22–23).

We must use bolder tools guided by a desire for structural solutions, collective care, reparative justice, liberation, and self-determination (Rakotovao et al., 2024). Challenging the narrative that racism does not live here in Canada is imperative. Recognizing the significance of systemic racism in everyday life opens space to uncover the ways in which various health disciplines and professions have been and remain complicit in promoting cultural and structural racism. Through this action, divesting from racism and investing in transformative solutions must follow.

Engaging in a critique of the taken-for-granted ways of doing will be necessary if we are to steer clear of “fantasy paradigms” that are common in action to reduce health inequities: that is, proposing solutions that are by no means up to the task while imagining that they are (Scott-Samuel & Smith, 2015). How we measure and understand the impact of racism matters. There are limits to simply naming the problem through its outcomes (e.g. collecting race-based data) without attending to the structures and policies that drive racial inequities. Agenor et al.’s database on state-level policies that are entangled in structural racism is a promising example to guide research and action (Agénor et al., 2021).

Public health must take an active role in advocating for healthy public policies that shift the distribution of power, resources, and wealth along intersectional racial lines. A focus on how racism influences all other determinants of health (e.g. income, labour market, education, transportation, food insecurity) should inform policies in sectors outside of health. For example, income and wealth, significant drivers of inequities, are highly racialized; yet, those policies have not typically considered how racism functions to generate inequities (Banting & Thompson, 2021). To reduce income and wealth inequities, policies must account for the role of racism. This includes large-scale placemaking to reduce racialized residential segregation and chronic disinvestment (Williams & Cooper, 2019).

Communities must be at the heart of change, and the law is a powerful tool for change. In 2024, the federal government passed the National Strategy Respecting Environmental Racism and Environmental Justice Act, which recognizes the importance of addressing environmental racism. This Act, which came to be through the advocacy of numerous researchers, communities, and organizations, demonstrates the value and power of collective action (The Canadian Coalition for Environmental & Climate Justice, 2022). The Toronto Black Food Sovereignty Plan, designed to improve food sovereignty for Black communities, is a “community led, municipally supported” plan that speaks to the responsibility of governments to address racial discrimination.

Addressing institutional racism in public health education through training new and existing population health actors in interdisciplinary approaches to dismantling structural racism, especially anti-Black racism and anti-Indigenous racism, is critical to providing researchers, practitioners, and policy makers with the tools needed (Alang et al., 2021; Ndumbe-Eyoh, 2020). To this end, the revised core competencies for public health include specific competencies on addressing anti-Black and anti-Indigenous racism, and the Black Health Education Collaborative is developing core public health competencies on Black health and addressing anti-Black racism.

## Conclusion

Racism and white supremacy rely on division, control, and dispossession. Decolonial anti-racism restores by recognizing the interdependence of all peoples and the places we inhabit. Strategies of (co-)resistance within and between communities centred in radical love and joy are necessary for Black communities to thrive. In community, we rest, we resist, we strategize, we thrive (hooks, 2003).

To achieve health equity, we must invest in the research, education, practice, and policy that are oriented towards understanding and dismantling racism. This includes investing at scale in systems of social care that address the cultural and structural drivers of racial inequities. Disrupting white supremacist norms and practices must infiltrate all aspects of health promotion, public, and population health. It is only through a comprehensive, decolonial, anti-racist framework that we can begin to address the deep-rooted health inequities faced by Black communities. To do so, we must contend with the historical and contemporary impacts of colonization and systemic anti-Black racism.

## Data Availability

N/A.

## Code availability

N/A.
